# CD47—a novel prognostic predicator in epithelial ovarian cancer and correlations with clinicopathological and gene mutation features

**DOI:** 10.1186/s12957-024-03308-6

**Published:** 2024-02-06

**Authors:** Xukai Luo, Jiahang Mo, Min Zhang, Wu Huang, Yiting Bao, Ruoyao Zou, Liangqing Yao, Lei Yuan

**Affiliations:** 1 Department of Gynecological Oncology, Obstetrics and Gynecology Hospital, Fudan University, Shanghai, 200011 China; 2 Institute of Reproduction and Development, Fudan University, Shanghai, 200011 China

**Keywords:** Epithelial ovarian cancer, Immunotherapy, CD47, Biomarker, Nomogram

## Abstract

**Background:**

Epithelial ovarian cancer (EOC) is insensitive to immunotherapy due to its poor immunogenicity; thus, suitable biomarkers need to be identified for better prognostic stratification and individualized treatment. CD47 is a novel immunotherapy target; however, its impact on EOC prognosis is controversial and correlation with genetic features is unclear. The aim of this study was to investigate the prognostic significance of CD47 and its correlations with biological behaviors and genetic features of EOC.

**Methods:**

Immunohistochemistry (IHC) and next-generation sequencing (NGS) were performed to examine expressions of CD47, PD-L1, and genomic mutations in the tissue samples of 75 EOC patients. Various clinicopathologic and genomic features were then evaluated to determine their correlation with CD47 expression. Kaplan–Meier analysis and Cox regression analysis were used to identify independent prognostic factors. Risk score modeling was then established, and the predictive capacity of this model was further confirmed by nomogram analysis.

**Results:**

CD47 was mainly expressed in the tumor cell membrane and cytoplasm, and the rate of high CD47 expression was 63.7%. CD47 expression was associated with various clinicopathological factors, including FIGO stage, CA125 and HE4 value, presence of multidisciplinary surgeries, presence and volume of ascites, lymph-node metastasis, Ki-67 index and platinum-resistant, as well as genetic characteristics like *BRCA* mutation, HRD status, and *TP53* mutation in EOC. Patients with high CD47 expression showed worse prognosis than the low-expression group. Cox regression analysis demonstrated that CA125, CD47, and *BRCA* mutation were independent factors for EOC prognosis. Patients were then categorized into high-risk and low-risk subgroups based on the risk score of the aforementioned independent factors, and the prognosis of the high-risk group was worse than those of the low-risk group. The nomogram showed adequate discrimination with a concordance index of 0.777 (95% CI, 0.732–0.822). The calibration curve showed good consistency.

**Conclusion:**

CD47 correlated with various malignant biology and genetic characteristics of EOC and may play pivotal and multifaceted roles in the tumor microenvironment of EOC Finally, we constructed a reliable prediction model centered on CD47 and integrated CA125 and *BRCA* to better guide high-risk population management.

**Supplementary Information:**

The online version contains supplementary material available at 10.1186/s12957-024-03308-6.

## Introduction

Ovarian cancer (OC) is known for extensive metastasis, common drug resistance, awful recurrence, and lethality, and epithelial ovarian cancer (EOC) is the most common pathologic type. Five-year survival for EOC remains suboptimal with available treatments [1, 2]. Recently, immunotherapy represented by immune checkpoint inhibitors (ICIs) has made remarkable progress in anti-tumor treatment [3]. PD-L1, the first-generation immune checkpoint (IC), and related inhibitors have been successfully approved for the treatments of various tumors [4, 5]. However, EOC is an immune-cold tumor leading to unsatisfactory therapeutic efficacy of available ICIs, and there are different reports on the prognosis of EOC with the expression pattern and level of PD-L1 [6, 7]. Nevertheless, the identification of effective immune targets for EOC has never stopped. Thus, understanding the mechanisms and interactions of immune components in the tumor microenvironment (TME) of EOC may be helpful for personalized medicine and prognosis improvement.

CD47 is identified as the first innate IC because of inhibition of macrophage phagocytosis by binding to signal regulatory protein α (SIRPα) [8]. Numerous evidences have demonstrated that CD47 was highly expressed in a variety of human malignancies, including OC, and was associated with poor prognosis [9, 10]. Moreover, blocking CD47-SIRPα signal showed remarkable anti-tumor effects in preclinical trials [11]. Although the effect of CD47 on the prognosis of EOC is partially controversial, CD47 is still regarded as a potential novel target considering its high expression profile in OC and promising preclinical results [12]. Additionally, EOC presents an obvious genetic predisposition, and *BRCA* mutation, a prognostic protective factor, is the most common genetic pathogenic gene. Patients with *BRCA* mutations or homologous recombination-deficient (HRD) can accept poly ADP-ribose polymerase inhibitors (PARPi) and significantly improve prognosis [13]. Unfortunately, PARPi is not approved for treatment in about half of the HR-proficient patients. Early studies have indicated that *BRCA* mutation is related with the number of tumor-infiltrating immune cells (TICs) in TME [11, 14]. Importantly, recent studies indicate that PARPi combined with anti-CD47 antibody showed significant anti-tumor effects in HR-proficient animal models [14, 15]. However, no real-world experience has simultaneously explored and supported the correlations of CD47 with related clinicopathological and genetic features in the TME of EOC, until now.

Here, we detected the expression of CD47 and related molecules, and gene mutations, with a particular focus on potential associations of CD47 with clinicopathological and genomic features, and prognosis of EOC. Meanwhile, we established a prognostic model in order to provide potential clues for prognostic stratification and individualized treatment of EOC in the future.

## Materials and methods

### Patients and tissue specimens

Formalin-fixed, paraffin-embedded 75 EOC tissue specimens were obtained from operations performed from 2017 to 2021 in the Gynecology and Obstetrics Hospital of Fudan University. All tissue specimens were collected under an Institutional Review Board-approved protocol. All tissue specimens obtained a final diagnosis and classification by specialists’ examination according to the International Federation of Gynecology and Obstetrics (FIGO, 2014). All patients were primary and excluded other gynecological malignancies. None of the patients received any form of chemotherapy, radiation, or immunotherapy prior to surgery. The clinicopathological information about the patients was collected from their clinical records and included their age, surgical stage, lymph-node metastasis, preoperative CA125 value, histological type, etc. After surgery, 74 patients received platinum- and paclitaxel-based first-line chemotherapy for 3–6 cycles according to National Comprehensive Cancer Network (NCCN) guidelines, except for one patient with IA-stage focal mucinous carcinoma. All patients received next-generation sequencing (NGS) after surgery. Thirty patients received PARPi maintenance therapy after first-line chemotherapy according to NCCN guideline. Optimal surgical debulking was defined as invisible residual lesion. Treatment response and relapse criteria were defined by the Response Evaluation Criteria in Solid Tumors guidelines version 1.1 (RECIST 1.1) and the Gynaecologic Cancer Intergroup (GCIG) CA125 definition [16, 17]. Post-treatment monitoring consisted of periodic clinical examination, serum tumor marker assay, and imaging evaluations. The progress-free survival (PFS) was defined as the time interval from diagnosis to disease progression, recurrence, or the most recent follow-up. The overall survival (OS) was defined as the time interval from diagnosis to the date of death or the most recent follow-up. No patient was lost to follow-up. Data were censored at the last follow-up for patients who had not relapsed or were still alive at the time of the analysis in May 2023.

### Immunohistochemistry (IHC)

The specimens were fixed in 10% neutral buffered formalin and subsequently embedded with paraffin. Histological sections of 5 mm were taken from each case of ovarian tissue. Each tissue had five serial sections. Sections were deparaffinized with xylene and rehydrated for further hematoxylin–eosin staining and IHC. The expression of CD47, PD-L1, and Ki-67 in EOC tissues was detected by IHC. Breast cancer, gastric cancer, and colon cancer tissues were used as positive controls for CD47, PD-L1, and Ki-67, respectively. Negative controls were incubated with phosphate buffers instead of primary antibodies. Four primary antibodies are anti-CD47 (Cat: ab218810, diluted 1:1000, Abcam), anti-PD-L1 (Cat: ab213524, diluted 1:100, Abcam), and anti-Ki-67 (Cat: ab16667, diluted 1:200, Abcam). The experimental procedure was performed in strict accordance with the manufacturer’s instructions.

### Assessment standard

IHC staining was scored by two independent experienced pathologists who were blinded to the clinical information of the patients. For CD47, staining localized to the cell membrane or cytoplasm was considered positive. According to staining, intensity was scored as 0, negative; 1, weak; 2, moderate; and 3, strong. According to the proportion of staining, area was scored as 0, 0%; 1, 1–9%; 2, 10–49%; 3, 50–79%; and 4, > 80%. Semi-quantitative score (range, 0–12) was obtained by multiplying the two scores together: 0 is considered ( −); 1 to 4 ( +); 6 to 8 (+ +); and 9 to 12 (+ + +).

According to the semi-quantitative score, the patients were divided into CD47 high expression group (+ + / +  + +) and CD47 low expression group (− / +). For PD-L1, the comprehensive positive score (CPS) was used, and CPS ≥ 1 was considered PD-L1 positive group. The assessment method of CPS was as follows: CPS = (*Number of tumor cells with positive PD-L1 membrane staining* + *Number of PD-L1 positive tumor-associated immune cells*) / *Total number of tumor cells* × *100* [18–20]. For Ki-67 index, nucleus-targeted staining is effective. According to the proportion of stained cells, the patients were divided into Ki-67 positive group (≥ 50%) and Ki-67 negative group (50% <).

### Next-generation sequencing (NGS)

NGS was analyzed with Oncoclear, a panel of 561 frequently mutated genes in OC developed by Precision Scientific (Beijing) Co., Ltd., Beijing, China. More details are as follows: (1) DNA Extraction: Primary tumor formalin-fixed, paraffin-embedded tissue samples and matched blood were used as the sources of genomic DNA. The ConcertBio HF system nucleus acid extract (Concert Bio) was used to extract genomic DNA. The Agilent 4200 (Agilent Technologies) and Qubit 4.0 (Thermo Fisher Scientific) were used to detect the DNA quality and quantity, respectively. (2) DNA Library Construction: Bioruptor (Diagenode) was used to shear 100 ~ 200 ng of DNA to short fragments (150 to 250 base pairs). These short fragments were repaired, and the two ends of the fragments were connected with adaptors. Selecting the DNA fragments of the targeted size. Next, a polymerase chain reaction was performed, and the resulting mixture was purified. The xGEN Prism DNA Library Prep Kit (Integrated DNA Technologies) was used to prepare sequencing libraries according to the manufacturer’s instructions. Hybridization enrichment was performed by probes targeting the corresponding panel, and Illumina NovaSeq 6000 was then used to sequenced validated DNA libraries. (3) Data analysis: Burrows-Wheeler Aligner (0.7.17) was used to map sequence data to the reference human genome. The Genome Analysis Tool Kit v.3.8.1 and VarScan v.2.4.3 were used to perform local alignment optimization and variant calling. VarScan fpfilter pipeline was used to filter variants, which loci with depth less than 100. Respectively, at least 8 and 5 supporting reads were required for single nucleotide variations and short insertion and deletion variations for base-calling of tissue samples.

### Statistical analysis

SPSS version 25.0 (SPSS Inc., Chicago, IL, USA) was performed for all statistical analyses. The relationships between the expression of targeted molecules and clinicopathological and genomic parameters were analyzed by *χ*^2^ test, modified *χ*^2^ test, or Fisher’s test. Kaplan–Meier (K-M) method was used to generate survival curves and compared by Log-rank test. Multivariate survival analysis of the prognostic factors was performed by Cox’s proportional hazard regression model. Statistically significant was defined as a bilateral *p* < 0.05.

### Development and assessment of a robust CD47-centered prognostic prediction model

R version 4.1.2 was performed for modeling and evaluation. In a multivariate COX regression model operation for the screened independent prognostic factors by R, the risk score was calculated according to the scores and regression coefficients of each influencing factor. All cases were then divided into high-risk and low-risk subgroups with a cut-off of 0 by the *Z*-score standardization of the scale function, and the survival differences between subgroups were analyzed by the K-M analysis. The predictive value of the prognostic model was evaluated by performing K-M analysis and nomogram construction. The “Survival” R package was performed for K-M analysis. Finally, the prediction nomogram was constructed with the screened independent prognostic factors, and then its predictive ability was judged by the corresponding calibration analysis.

## Results

### Study population

The patients’ clinicopathological characteristics were presented in Table 1. The median age of the study population was 51 years (range 28–72 years). Most patients are presented at FIGO stage III (36/75, 48.0%), with serous tumors (50/75, 66.7%), and with complete response to frontline therapy (62/75, 92.0%). Majority of patients (60/75, 80.0%) underwent optimal debulking surgery, and a positive Ki-67 index was presented in 41 (54.7%) cases **(**Supplementary Fig. 1).

**Table 1:** Patient characteristics

Characteristics, number (%)	*n* = 75 (100)
Age, years	
Median	51.0
Range	28–72
FIGO stage	
I	18 (24.0)
II	5 (6.7)
III	36 (48.0)
IV	16 (21.3)
Histological type	
Serous	50 (66.7)
Endometroid	9 (12.0)
Mucinous	8 (10.7)
Clear cell	6 (8.0)
Mixed	2 (2.6)
Preoperative serum CA125	
< 500 U/mL	42 (56.0)
≥ 500 U/mL	33 (44.0)
Preoperative serum HE4	
Normal^a^	17 (22.7)
Increased	52 (69.3)
Unknown	6 (8.0)
Multidisciplinary surgeries	
No	33 (44.0)
Yes	42 (56.0)
Ascites	
None	18 (24.0)
< 1000 ml	36 (48.0)
≥ 1000 ml	21 (28.0)
Lymph-node metastasis	
Negative	37 (49.3)
Positive	38 (50.7)
Residual lesion	
Optimal (*R*_0_)	60 (80.0)
Suboptimal (*R*_1_ + *R*_2_)	15 (20.0)
Ki-67 index, number (%)	
Negative	34 (45.3)
Positive	41 (54.7)
Response to frontline therapy	
Complete response	69 (92.0)
Partial response	3 (4.0)
Progression disease	3 (4.0)
PARPi maintenance	
No	45 (60.0)
Yes	30 (40.0)
Platinum status	
Sensitive	62 (82.9)
Resistant	13 (17.1)

^a^Normal reference range of HE4: < 40 years, < 60.5; 40–49 years, < 76.2; 50–59 years, < 74.3; 10–69 years, < 82.9; > 70 years, < 104 pmol/L

### The result of the NGS

The top ten frequently mutated genes and HRD and microsatellite (MSI) status of the study population were presented in Table 2. *TP53*, the most mutated gene, was found in 54 (72.0%) cases, and patients with *BRCA1* and *BRCA2* mutation were found in 15 (20.0%) and 8 (10.7%) cases, respectively. Twenty patients were not detected HRD status due to HRD testing was not widely available and expensive in earlier years, and 35 patients in the tested population were HRD positive. For MSI status, the majority of patients presented microsatellite stability (MSS)/MSI-low (70/75, 93.3%).

**Table 2:** Next-generation sequencing: the top ten most frequently mutated genes and HRD and MSI status in EOC patients

Genes, number (%)	
* TP53*	54 (72.0)
* BRCA1*	15 (20.0)
* PIK3CA*	13 (17.3)
* BRCA2*	8 (10.7)
* MYC*	9 (12.0)
* KRAS*	8 (10.7)
* NF1*	8 (10.7)
* ARID1A*	7 (9.3)
* CDKN2A*	6 (8.0)
* PMS2*	6 (8.0)
HRD status^a^, number (%)	
Negative	20 (39.4)
Positive	35 (63.6)
MSI status, number (%)	
MSS/MSI-L	70 (93.3)
MSI-H	5 (6.7)

^a^HRD status was not detected in 20 patients

### Expression of CD47 and PD-L1 in EOC

**Fig. 1:**
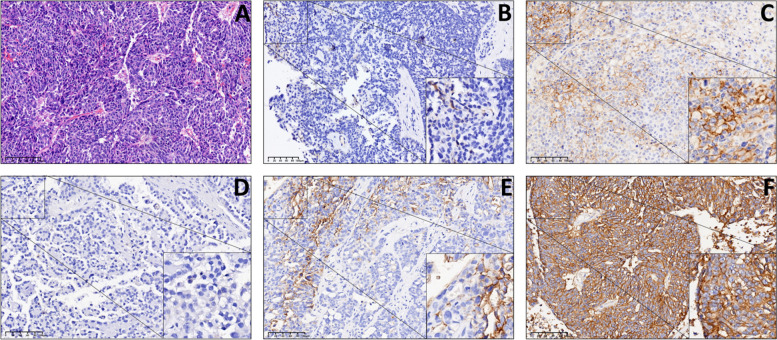
CD47 and PD-L1 expression in EOC by immunohistochemistry and Hematoxylin–Eosin (200 ×). **A** HE staining was used for verification. **B** Negative expression (CPS < 1) of PD-L1. **C** Positive expression (CPS = 35) of PD-L1. **D** Negative expression (0, −) of CD47. **E** Low expression (2, +) of CD47. **F** High expression (12, +  + +) of CD47

CD47 protein was expressed in the cell membrane and cytoplasm of EOC samples and mainly localized on the former. Positive and high CD47 expression were presented in 72 (96.0%) and 47 (63.7%) cases, respectively. For PD-L1, its expression profile was different from CD47, and its positive rate was only 36% (27/75 cases) in the study population. Notably, PD-L1 was mainly expressed on tumor-associated immune cells rather than tumor cells (Fig. 1 and Table 3).

**Table 3:** Expression of CD47 and PD-L1 in EOC

Groups	Cases	−	+	+ +	+ + +	Positive cases	Positive rate (%)	High-expression cases	High-expression rate (%)
CD47	75	3	25	21	26	72	96.0	47	63.7
PD-L1	75	48	27^a^			27	36.0	27	36.0

^a^The positive expression of PD-L1 was not further distinguished by the degree of expression

### Relationships of CD47 expression with the clinicopathological and genomic characteristics of EOC

High CD47 expression was significantly associated with multiple high-risk clinicopathological and genomic characteristics including advanced FIGO stage (*p* < 0.001), high preoperative serum CA125 and HE4 value (*p* = 0.002, *p* = 0.013), presence of multidisciplinary surgeries (*p* < 0.001), presence and massive volume of ascites (*p* = 0.003, *p* < 0.001), positive lymph-node metastasis (*p* < 0.001), positive Ki-67 index (*p* = 0.002), and pathogenic *TP53* mutation (*p* = 0.001). Importantly, there was a significant correlation between high CD47 expression and poor chemotherapy response (*p* = 0.002), and all the 13 platinum-resistant patients presented high CD47 expression. In contrast, high CD47 expression was also associated with positive HRD status and pathogenic *BRCA* mutation (*p* = 0.003, *p* = 0.021), two prognostic protective factors in EOC. Additionally, high CD47 expression was associated with positive PD-L1 expression (*p* = 0.014). No statistical difference was found between CD47 expression, and residual lesion, MSI status and response to frontline therapy, all the *p* values > 0.05. Notably, all the patients in the partial response (PR) or progress disease (PD) group presented high CD47 expression (6/6 cases, 100%) (Table 4).

**Table 4:** Relationship between CD47 and clinicopathological and genomic characteristics of EOC

Characteristics	Cases	CD47 expression		*p*-value
		Low [cases (%)]	High [cases (%)]	*-*
FIGO stage	75	-	-	< 0.001
I/II	23	19 (82.6)	4 (17.4)	-
III/IV	52	9 (17.3)	43 (82.7)	-
Preoperative serum CA125	75	-	-	0.002
< 500 U/mL	42	22 (52.4)	20 (47.6)	-
≥ 500 U/mL	33	6 (18.2)	27 (81.8)	-
Preoperative serum HE4	69^a^	-	-	0.013
Normal	17	11 (64.7)	6 (35.3)	-
Increased	52	16 (30.8)	36 (69.2)	-
Multidisciplinary surgeries^b^	75	-	-	< 0.001
No	33	26 (78.8)	7 (21.2)	-
Yes	42	2 (4.8)	40 (95.2)	-
Ascites	75	-	-	0.003^▲^ 0.010^▲▲^
None	18	12 (66.7)	6 (33.3)	-
< 1000 ml	36	13 (36.1)	23 (63.9)	-
≥ 1000 ml	21	3 (14.3)	18 (85.7)	-
Lymph-node metastasis	75	-	-	< 0.001
Negative	37	25 (67.6)	12 (32.4)	-
Positive	38	3 (7.9)	35 (92.1)	-
Ki-67 index	75	-	-	0.002
Negative	34	19 (55.9)	15 (44.1)	-
Positive	41	9 (22.0)	32 (78.0)	-
PD-L1 expression	75	-	-	0.014
Negative	48	23 (47.9)	25 (52.1)	-
Positive	27	5 (18.5)	22 (81.5)	-
Residual lesion	75	-	-	0.753
Optimal	60	23(38.3)	37(61.7)	-
Suboptimal	15	5(33.3)	10(66.7)	-
MSI status	75	-	-	0.407
MSS/MSS-L	70	27 (38.6)	43 (61.4)	-
MSS-H	5	1 (20.0)	4 (80.0)	-
HRD status	55^†^	-	-	0.003
Negative	20	11 (55.0)	9 (45.0)	-
Positive	35	6 (17.1)	29 (82.9)	-
*BRCA* mutation status	75	-	-	0.021
Wild type	52	24 (46.2)	28 (53.8)	-
Pathogenic mutation	23	4 (17.4)	19 (82.6)	-
*TP53* mutation status	75	-	-	0.001
Wild type	21	14 (66.7)	7 (33.3)	-
Pathogenic mutation	54	14 (25.9)	40 (74.1)	-
Response to frontline therapy	75	-	-	0.126
CR^†^	69	28 (40.6)	41 (59.4)	-
PR or PD^††^	6	0 (0.0)	6 (100.0)	-
Platinum status	75	-	-	0.002
Sensitive	62	28 (45.2)	34 (54.8)	-
Resistant	13	0 (0.0)	13 (100.0)	-

^▲^Both ascites < 1000 ml and ascites ≥ 1000 ml were used as control group

^▲▲^Both ascites < 1000 ml and none ascites were used as control group

^†^HRD status was not detected in 20 patients

^†^*CR*, complete response

^††^*PR*, partial response; *PD*, progress disease

^a^Preoperative serum HE4 values were missing in six patients

^b^Multidisciplinary surgeries are defined as those that transcend the scope of gynecology, such as liver resection, spleen resection, and bowel resection

### Correlations of CD47 expression and related clinicopathological and genomic characteristics with clinical prognosis

**Fig. 2:**
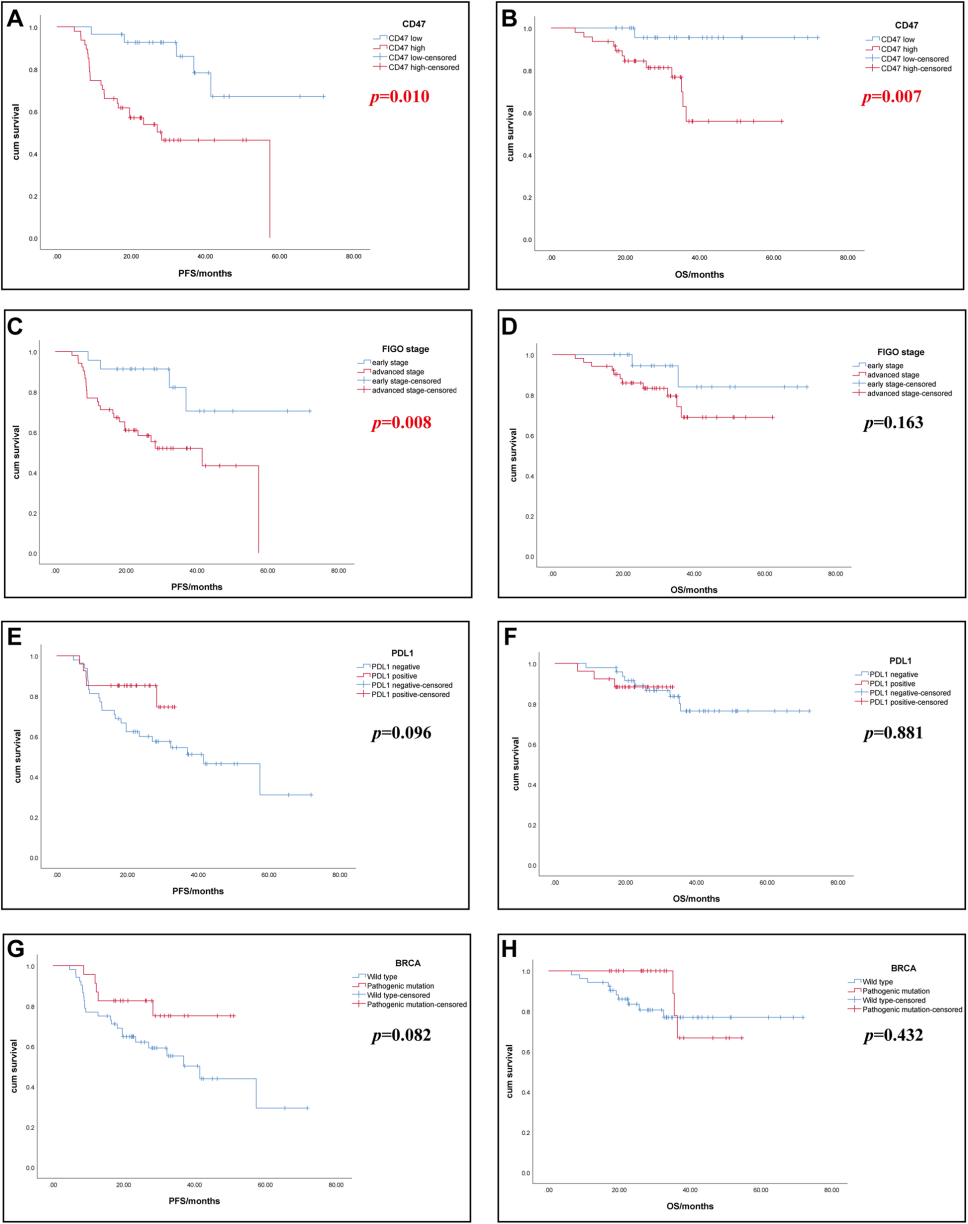
K-M analyses for study population according to CD47, PD-L1 expression, FIGO stage, and *BRCA* mutation. **A** PFS by CD47 expression categorized as high expression vs. low expression. **B** OS by CD47 expression categorized as high expression vs. low expression. **C** PFS by FIGO stage categorized as advanced stage vs. early stage. **D** OS by FIGO stage categorized as advanced stage vs. early stage. **E** PFS by PD-L1 expression categorized as positive expression vs. negative expression. **F** OS by PD-L1 expression categorized as positive expression vs. negative expression. **G** PFS by *BRCA* mutation status categorized as mutated type vs. wild type. **H** OS by *BRCA* mutation status categorized as mutated type vs. wild type

K-M curves showed that PFS (*p* = 0.010) and OS (*p* = 0.007) significantly decreased in patients with high CD47 expression (Fig. 2A and B). The median PFS of the high CD47 expression group was 28.2 months, while the median PFS of the low CD47 expression group was not reached. Results also showed that the advanced FIGO stage group with a shorter PFS (*p* = 0.008), and the median PFS was 41.5 months (Fig. 2C). Although no significant difference was found in OS (*p* = 0.163) between the different FIGO stage groups, the K-M curves showed a trend towards worse OS in the advanced stage group. It was possibly due to insufficient follow-up time (Fig. 2D). Considering the specific expression pattern of PD-L1 in our study population and its controversial prognostic impact on EOC, PD-L1 was included as a dependent variable in the K-M analysis. However, no significant difference was found in prognosis between positive and negative PD-L1 groups, but long-term PFS seemingly appeared to differ (Fig. 2E and F). Therefore, further K-M analysis showed that the PD-L1 negative group with a significant shorter PFS (*p* = 0.007) from 8.4 months onwards, and the median PFS was 41.5 months (Supplementary Fig. 2). Similarly, no significant difference was found in prognosis (*p* = 0.082 and *p* = 0.432) between the different *BRCA* mutation groups, but K-M curves showed a trend towards worse PFS in the *BRCA*-wild type group (Fig. 2G and H).

### Identification of prognostic factors and establishment of related prediction model

**Fig. 3:**
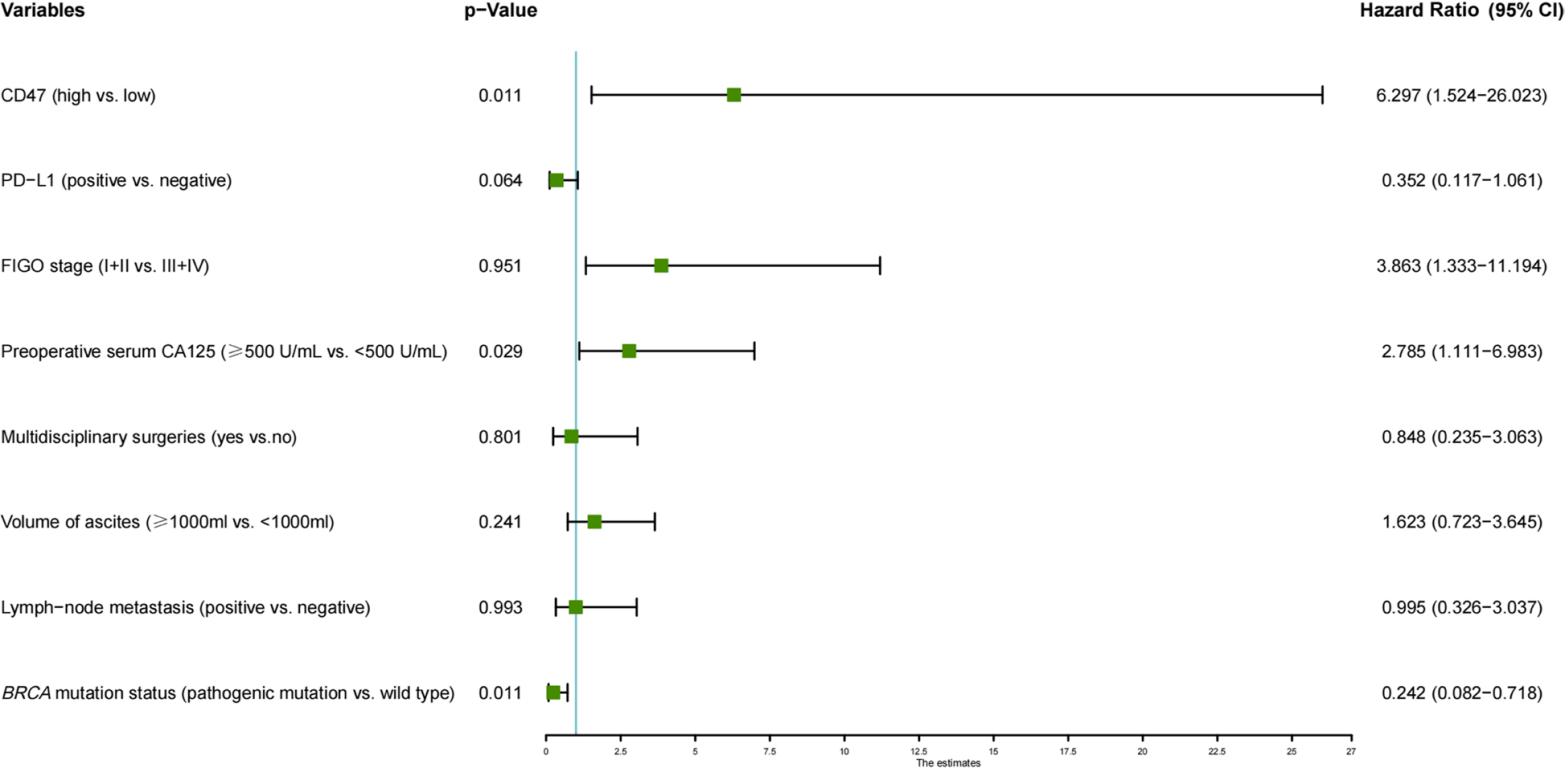
Multivariate analysis of the PFS in EOC compared with clinicopathological and genomic characteristics and CD47. Dependent variables included CD47, PD-L1, FIGO stage, preoperative serum CA125 value, presence of multidisciplinary surgeries, volume of ascites, lymph-node metastasis, and *BRCA* mutation status

In univariate analysis, CD47 expression, FIGO stage, preoperative serum CA125, multidisciplinary surgeries, volume of ascites, and lymph-node metastasis were associated with PFS (Table 5). PD-L1 expression and *BRCA* mutation status were also included in the multivariate analysis along with the above meaningful variables in the univariate analysis because of their potential impact on prognosis demonstrated by the K-M curves. The results showed that CD47 expression and CA125 value were independent risk factors for PFS time of EOC (HR = 6.297, *p* = 0.011; HR = 2.785, *p* = 0.029). As expected, *BRCA* mutation known as EOC prognostic protective factor was reverified (HR = 0.242, *p* = 0.023). PD-L1 expression showed potential as a prognostic protective factor for EOC (HR = 0.352, *p* = 0.064) (Fig. 3).

**Table 5:** Univariate analyses of the PFS in EOC compared with clinicopathological and genomic characteristics and CD47

Variables	*p*-value	Hazard ratio (95% CI)
CD47 (high vs. low)	0.002	4.449 (1.698–11.924)
PD-L1 (positive vs. negative)	0.105	0.445 (0.167–1.183)
FIGO stage (I + II vs. III + IV)	0.013	3.863 (1.333–11.194)
Preoperative serum CA125 (≥ 500 U/mL vs. < 500 U/mL)	0.015	2.573 (1.198–5.525)
Preoperative serum HE4 (increased vs. normal)	0.466	1.451 (0.533–3.949)
Multidisciplinary surgeries (yes vs.no)	0.006	3.173 (1.386–7.261)
Presence of ascites (yes vs.no)	0.135	2.242 (0.779–6.453)
Volume of ascites (≥ 1000 ml vs. < 1000 ml)	0.037	2.201 (1.049–4.620)
Lymph-node metastasis (positive vs. negative)	0.005	3.110 (1.404–6.890)
Ki-67 index (positive vs. negative)	0.129	1.800 (0.843–3.845)
Residual disease (optimal vs. suboptimal)	0.544	1.302 (0.555–3.057)
*BRCA* mutation status (pathogenic mutation vs. wild type)	0.091	0.434 (0.165–1.142)
HRD status^a^ (positive vs. negative)	0.074	0.429 (0.169–1.087)
*TP53* mutation status (pathogenic mutation vs. wild type)	0.877	1.065 (0.476–2.383)

^a^K-M curves showed similar results to *BRCA* mutation, and HRD status was not included in subsequent multivariate analysis due to the complete *BRCA* mutation was contained in HRD status (Supplementary Fig. 3)

**Fig. 4:**
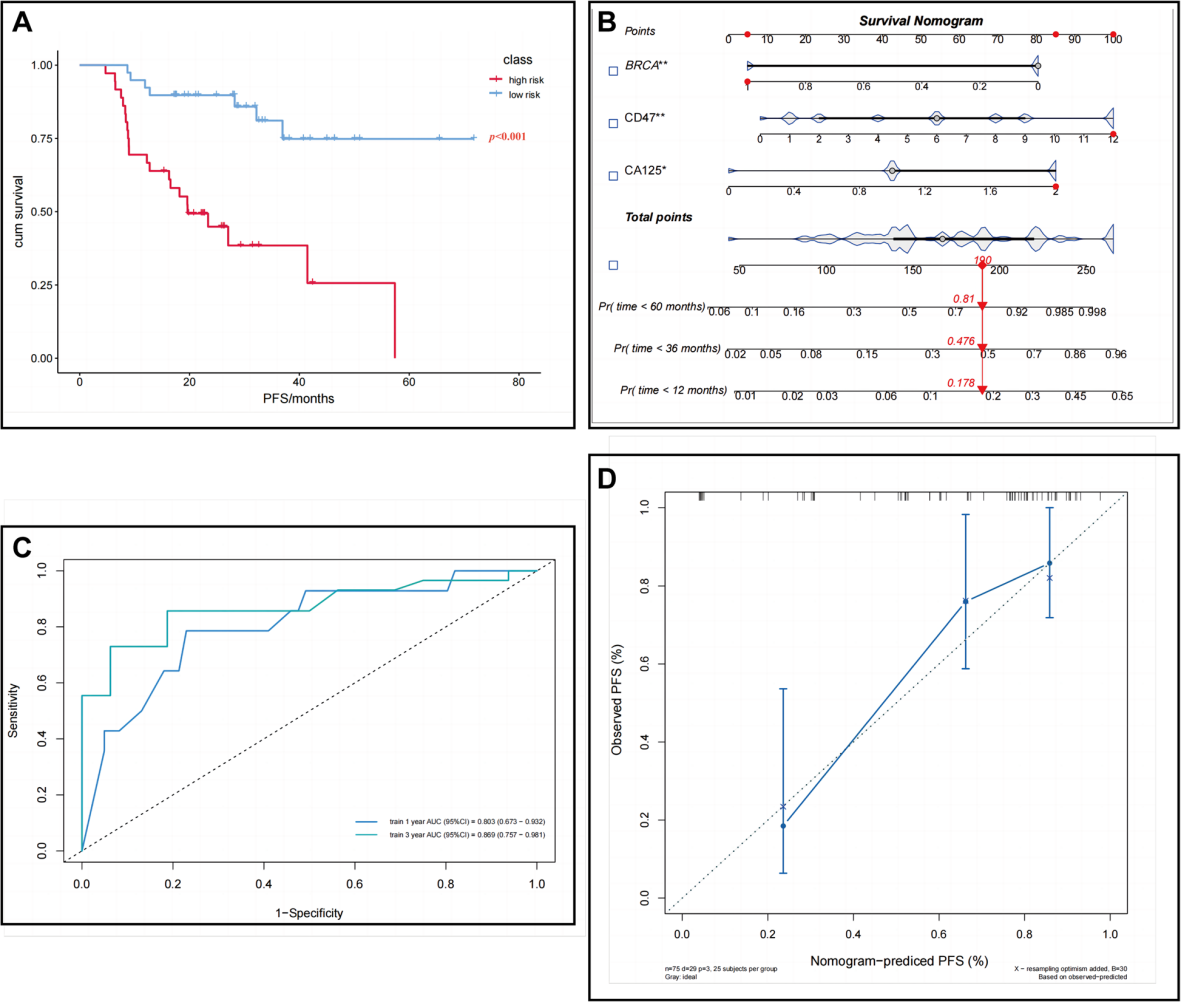
Survival analysis, and the nomogram for predicting the probability of patients’ recurrence rate. **A** K-M survival analyses for the PFS of patients between the high-risk and low-risk subgroups. **B** Nomogram plots for predicting the probability of recurrence rate in study cohort based on risk score and independent prognostic factors. **C** The AUC of ROC curve for the nomogram used to predict the 1- and 3-year PFS for our study population was satisfactory (0.803, 95% CI, 0.673 to 0.932; 0.869, 95% CI, 0.757 to 0.981). **D** Calibration plot depicted good agreement between nomagram predicted and observed 3-year PFS, representing the ideal predictions

CD47 expression, CA125 value, and *BRCA* mutation were used to construct a predictive model based on multifactorial COX results. CA125 value was delineated interval, and CD47 expression was adapted IHC semi-quantitative score for modeling rather than binary data in order to better differentiate and increase the suitability for clinical applications, and the specific modified data were shown in Supplementary Table 1. Consequently, a prognosis prediction model centered on CD47 was constructed by the adjusted data following the formula: risk score = (0.159×CD47 expression) + (0.884×CA125 value) + (− 1.570×*BRCA* mutation). The K-M analysis of our study population showed that PFS was significantly worse in the high-risk group than in the low-risk group (Fig. 4A). Subsequently, the above data were utilized to create a nomogram for predicting 1-, 3-, and 5-year PFS. The concordance index for this nomogram achieved 0.777 (95% CI, 0.732–0.822) (Fig. 4B). Our nomogram area under the curve (AUC) used to predict the 1-year and 3-year PFS achieved 0.803 (95% CI, 0.673–0.932) and 0.869 (95% CI, 0.757–0.981), respectively (Fig. 4C). Calibration curve for the probability of 3-year PFS showed optimal agreement between the predicted and actual probabilities (Fig. 4D).

## Discussion

CD47 had become a star anti-tumor molecule since its immune checkpoint (IC) identity was revealed [12]. Initially, CD47 was known as integrin-associated protein involved in inflammatory cell chemotaxis, adhesion, migration, and activation of anti-inflammatory response and platelets. Its diverse biological functions and ligands were gradually discovered with further researches. In addition to regulating cell proliferation, apoptosis, adhesion, and migration, CD47 binding to thromboporetin-1 is also involved in angiogenesis and perfusion. Connection of CD47 and SIRPα could transmit inhibitory signals to prohibit macrophage phagocytosis, leading to immune evasion of tumor cells and ultimately promoting tumor development. The unique macrophage inhibitory property of CD47, resulting CD47 becoming the first identified innate IC and regarding as another research hotspot after PD-L1 [21–24]. Researches have demonstrated that CD47 was highly expressed in various human malignancies, including EOC, and was associated with various adverse clinicopathological factors. An association was found between CD47 overexpression and poor prognosis, including acute myeloid leukemia and non-Hodgkin’s lymphoma [25, 26]. However, the impact of CD47 on OC prognosis is currently debated. In most studies, CD47 was an independent risk factor for poor prognosis of OC [27, 28]. Our results confirmed the observation of the most previously reported studies that high CD47 expression was associated to a poor prognosis, as well as the significant correlations with clinicopathological features, including advanced FIGO stage, lymph-node metastasis, chemotherapy resistance, and high serum CA125 value. Intriguing, all the 13 platinum-resistant patients present high CD47 expression in our study. Similarly, further analysis showed that the high CD47 expression was also significantly (*p* = 0.027) correlated with platinum resistance in the recurrence subgroup (Supplementary Table 2). It implies that CD47 is valuable for predicting platinum resistance. Cancer stem cell (CSC) represents a subset of tumors endowed with the ability to initiate tumor progression, metastasis, recurrence, and drug resistance. Chang et al. found OC stem-like cells could be protected from immune attack by surrounding non-stem cell cancer cells with low CD47 expression. Obviously, these escaped cells play vital roles in recurrence [29, 30]. Hyperactivation of the phosphatidylinositol 3-kinase/protein kinase B/mammalian target of rapamycin (PI3K/AKT/mTOR) pathway is one of the common mechanisms leading to tumor resistance. CD47 has been confirmed to promote the invasion of glioblastoma and endometrial carcinoma cells via the PI3K/AKT/mTOR-mediated glycolysis pathway [31, 32]. Pai and Zhang et al. demonstrated that knockdown of *CD47* gene in oral squamous cell carcinoma and hepatocellular carcinoma could down-regulate the resistance caused by the above glycolytic pathway to improve therapeutic efficacy, respectively [33, 34]. There is still a gap in this area for OC, and further research may help to figure out the dilemma of OC resistance. Additionally, attributable to the comprehensive clinicopathological, genomic, and follow-up data, we firstly found that high CD47 expression was also associated with increased serum HE value, presence and volume of ascites, pathogenic *TP53* mutation, and positive Ki-67 index in EOC. It was worth mentioning that the relationships between these factors and the occurrence and development of EOC were clear, such as positive Ki-67 index associated with tumor proliferation and *TP53* mutated with various tumorigenesis and invasion. These results indicated that CD47 was closely related to the multiple aspects of occurrence and development in EOC, including proliferation, invasion, metastasis, and drug resistance.

Brightwell et al. reported there were no differences in OC survival for different CD47 expressions, neither in their exploratory analysis of the Cancer Genome Atlas (TCGA) data nor subsequent retrospective study. Importantly, this was the largest retrospective study to date on the effect of CD47 in prognosis in a population with OC. Low CD47 expression was associated with better chemotherapy response in their study but did not translate into better prognosis. Brightwell explained this unusual result for OC with complex immune heterogeneity, and CD47 likely operated in concert with other immune escape mechanisms, these potential pathways included PD-1/PD-L1, LAG-3, and Tregs [35]. In addition, we considered that Brightwell defined high and low CD47 groups in the TCGA data based on the presence of gene mutations rather than CD47 protein expression levels may also contributed to this unusual result, as well as uncommon immunohistochemical score in their retrospective study. Combined with the conclusions of Brightwell and our study, high CD47 expression was associated with HRD positive and *BRCA* and *TP53* mutations, we speculated that this population might benefit more from relevant targeted therapy and immunotherapy, whereas the low expression group preferred chemotherapy (Supplementary Fig. 5). Needless to say, further researches are needed to confirm these hypotheses.

PD-L1 used to be considered a predictive molecule and target for anti-tumor immunotherapy, whereas now, its prognostic significance is controversial. PD-L1 could inhibit T cell activation and tumor adaptive immunity, and was associated with poor prognosis. Recent studies have shown that PD-L1 was primarily expressed by tumor-associated macrophages (TMA), causing an association with better survival in EOC [6]. Indeed, similar conclusions had been illustrated earlier in other tumors [7]. In line with other studies, we observed that PD-L1 mainly expressed on TICs, and such expression pattern presented a potential long-dated protective effect (Fig. 1, Supplementary Figs. 2 and 4). Adaptive immunity may play a key role in this prognostic-protective effect, including activation and increased levels of cytotoxic T cells and TICs [36, 37]. Previous studies have demonstrated that simultaneously high CD47 and positive PD-L1 expression on tumor cells could synergistically promote tumor development, and that dual-blockade could achieve better anti-tumor effects than single blockade of CD47 or PD-L1 [12, 38]. Our study might reveal a novel and more common synergistic pro-tumor immunophenotyping. First, negative PD-L1 expression on TICs was related to suppression of adaptive immunity as mentioned [36, 37]. Second, CD47-SIRPα signal both inhibits the innate immunity by suppression of macrophage phagocytosis and NK cells’ killing effects, and the adaptive immunity by suppression of tumor antigens cross-presentation and T cell activation [12, 38–40]. The combinations of both effects created a fully immunosuppressive TME promoting EOC development. Perhaps, it is the unique expression pattern of PD-L1 that leads to the poor efficacy of PD-L1/PD-1 inhibitors in EOC [18, 41]. On the contrary, the high expression and clear biological functions of CD47 in EOC make it a more suitable therapeutic and prognostic target compared to PD-L1.

*BRCA* pathogenic mutation presented in 30.7% (23/75) of patients in our study, similar to the frequency reported in the Chinese population [13, 42]. Consistent with previous studies, *BRCA* mutation was a protective factor for EOC prognosis in our study (Fig. 4). Although we observed that *BRCA* pathogenic mutation was associated with high CD47 expression, the hazard ratio (HR) of CD47 was significantly greater than that of *BRCA* mutation status, indicating that CD47 could cover the former’s protective effect (Fig. 4). A series of studies demonstrated that *BRCA* mutation and HRD status could influence prognosis through tumor immunity, such as patients with *BRCA* mutation or HRD positive had increased TICs in TME, especially CD8^+^ T cells [43–45]. PARPi was approved for the maintenance treatment of OC with *BRCA* mutation or HRD. On the contrary, Al-Sudani et al. recently observed the combination of anti-CD47 antibody with PARPi led to significant anti-tumor effects via enhanced in vitro phagocytosis and Sting pathways in *BRCA*-wild OC patient-derived xenograft model [15]. Subsequently, Liu et al. demonstrated Olaparib increased the numbers and phagocytosis of TAM in TME via partial dependence on the CD47-SIRPα signal in HRD-negative mouse model. Likewise, the concomitant administration of Olaparib with anti-CD47 antibody amplified TAM-mediated tumor regression than monotherapy [14]. The subgroup with the largest proportion in our study, CD47-high + *BRCA-*wild, presented the worst prognosis (the median PFS, 16.5 months) compared with other subgroups and patients with high CD47 expression (28.2 months) or *BRCA* wild type (41.5 months) alone (Supplementary Fig. 4). Of patients, 78.3% (18/23) with *BRCA* mutation received PARPi in our study, so these results well explain why the CD47-high + *BRCA-*wild subgroup presented the worst prognosis. Importantly, these studies provided theoretical basis for expanding the indications of PARPi in OC patients, and provided possibilities of novel combined therapy for high-risk group.

Finally, a novel and accurate prognostic prediction model consisting of three predictors (CA125 value, CD47 expression, and *BRCA* mutation) was established. Notably, our nomogram area under the curve (AUC) (0.803, 95% CI, 0.673–0.932; 0.869, 95% CI, 0.757–0.981) (Fig. 4C) used to predict 1- and 3-year PFS for EOC patients was significantly higher than the AUC of other nomograms [46, 47]. On the basis of the existing necessary detection items of EOC, incorporating easy-to-detect CD47 IHC score makes our model low-cost and convenient application.

Our study population was from a single center, and the number was relatively small, resulting in a relatively poor representation of statistical analysis results. Studies in a multicenter and larger EOC cohort and other histological OC cohort are necessary to validate the predictive efficiency of CD47 and related model. The censored HRD data led to *BRCA* mutation as one of the main subjects for subsequent analyses. In fact, HRD status is applicable to a wider patient population; it is necessary to analyze the HRD status for subsequent studies. OS was not the primary endpoint in our study due to the limited follow-up time, and subsequently, close follow-up of this patient population was necessary to explore the predictive values of these above molecules on OS. Moreover, although semi-quantitative scoring is the most classical and widely used immunohistochemical scoring modality, it is undeniable that current image capture and artificial intelligence technologies may be able to provide a more accurate and uniform criteria.

Up to now, PD-1/PD-L1 inhibitors and PARPi were approved for the treatment of breast cancer, OC, prostate cancer, and leukemia [14, 48]. CD47-targeted drugs are in development and demonstrate certain anti-tumor activities and can improve prognosis in clinical trials. Various CD47-related drugs showed promising activity and well-tolerated safety events in phase I and II clinical trials, thus Phase III clinical trials are currently in full swing, but no official data has been published [12]. Our study reveals that CD47 affected the biological behaviors of EOC in multiple aspects, and it was an independent and robust predictor of prognosis. Moreover, preclinical studies suggest that combination therapy can improve efficacy and hopefully solve the treatment dilemma of OC [49].

## Conclusion

Summarily, our study indicates that CD47 plays pivotal and multifaceted roles during multiple biological processes in the TME of EOC, implying its potential to be a stable prognostic predictor and a promising therapeutic target. Finally, we constructed a reliable prediction model centered on CD47 and integrated CA125 and *BRCA* for accurate prognosis prediction and individualized medicine in OC.

### Supplementary Information

**Additional file 1:** **Supplementary Fig. 1.** Ki-67 index in epithelial ovarian cancer by immunohistochemistry staining (200×). A Negative Ki-67 index (15%). B Positive Ki-67 index (50%). (C) Positive Ki-67 index (80%).

**Additional file 2: Supplementary Fig. 2.** K-M analysis for study population according to PD-L1 expression after 8.4 months.

**Additional file 3:** **Supplementary Fig. 3.** K-M analysis for study population according to HRD status. (A) PFS by HRD status categorized as positive vs. negative. (B) OS by HRD status categorized as positive vs. negative.

**Additional file 4:** **Supplementary Fig. 4.** K-M analysis for the subgroup according to the combination of CD47 expression and *BRCA* mutation. PFS by the combination of CD47 expression and *BRCA* mutation status categorized as CD47-high+*BRCA*-wild subgroup vs. CD47-low+*BRCA*-mutated, p=0.470; CD47-low+*BRCA*-wild, p<0.001; and CD47-high+*BRCA*-mutated subgroups, p=0.006, respectively.

**Additional file 5:** **Supplementary Fig. 5. **Possible efficacy therapies in different CD47 expression group.

**Additional file 6:** **Supplementary Table 1. **Patients’ IHC semi-quantitative score of CD47 expression, CA125 range intervals, and *BRCA* mutation status. **Supplementary Table 2. **Relationship between CD47 and platinum status in recurrent subgroup.

## Data Availability

All data generated or analyzed during this study are included in this published article.

## Abbreviations

CPS: Comprehensive positive score
CSC: Cancer stem cell
EOC: Epithelial ovarian cancer
GCIG: Gynaecologic cancer intergroup
HRD: Homologous recombination-deficient
IC: Immune checkpoint
ICIs: Immune checkpoint inhibitors
IHC: Immunohistochemistry
K-M: Kaplan–Meier
MSI: Microsatellite
NCCN: National Comprehensive Cancer Network
NGS: Next-generation sequencing
OC: Ovarian cancer
OS: Overall survival
PARPi: Poly ADP-ribose polymerase inhibitors
PFS: Progress-free survival
PI3K/AKT/mTOR: Phosphatidylinositol 3-kinase/protein kinase B/mammalian target of rapamycin
RECIST: Response evaluation criteria in solid tumors
SIRPα: Signal regulatory protein α
TCGA: The cancer genome atlas
TICs: Tumor-infiltrating immune cells
TMA: Tumor-associated macrophages
TME: Tumor microenvironment
