# Supplementary figures and images for: CD47—a novel prognostic predicator in epithelial ovarian cancer and correlations with clinicopathological and gene mutation features
Source: World J Surg Oncol. 2024 Feb 6;22:44. doi: 10.1186/s12957-024-03308-6 (PMC10845810; doi:10.1186/s12957-024-03308-6)

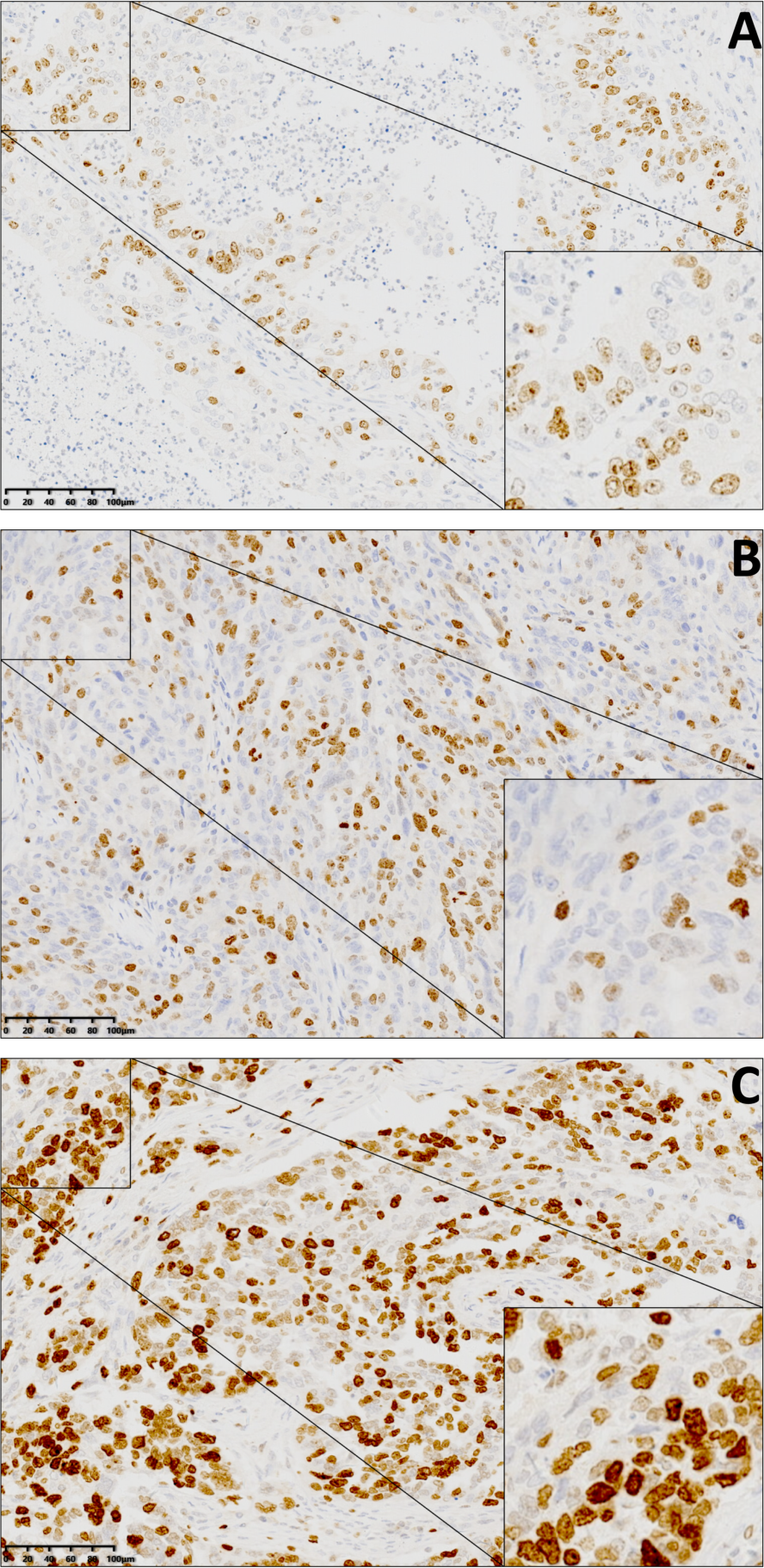

Supplement: Supplementary file 1 — Additional file 1: Supplementary Fig. 1. Ki-67 index in epithelial ovarian cancer by immunohistochemistry staining (200×). A Negative Ki-67 index (15%). B Positive Ki-67 index (50%). (C) Positive Ki-67 index (80%). [file 12957_2024_3308_MOESM1_ESM.tif]

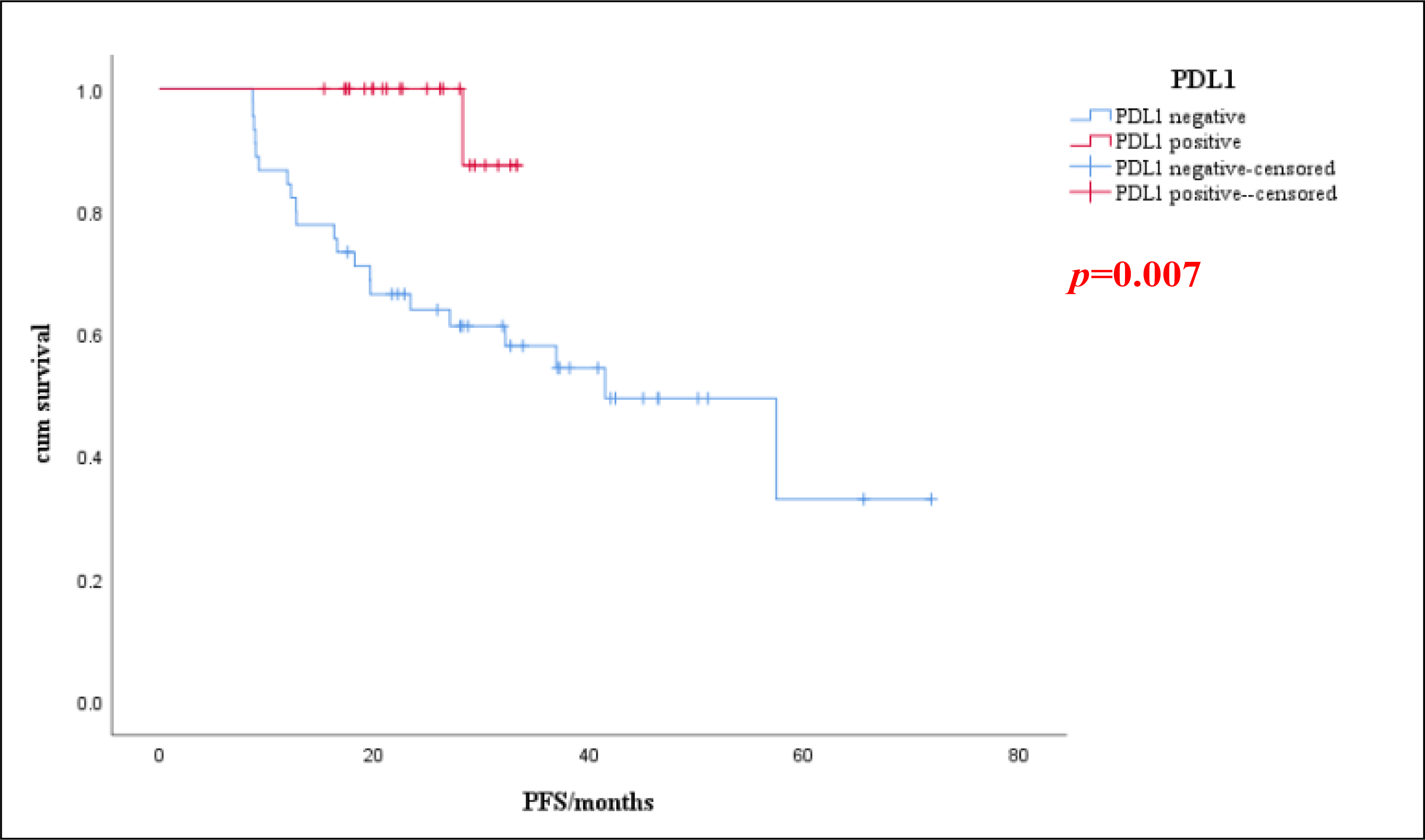

Supplement: Supplementary file 2 — Additional file 2: Supplementary Fig. 2. K-M analysis for study population according to PD-L1 expression after 8.4 months. [file 12957_2024_3308_MOESM2_ESM.tif]

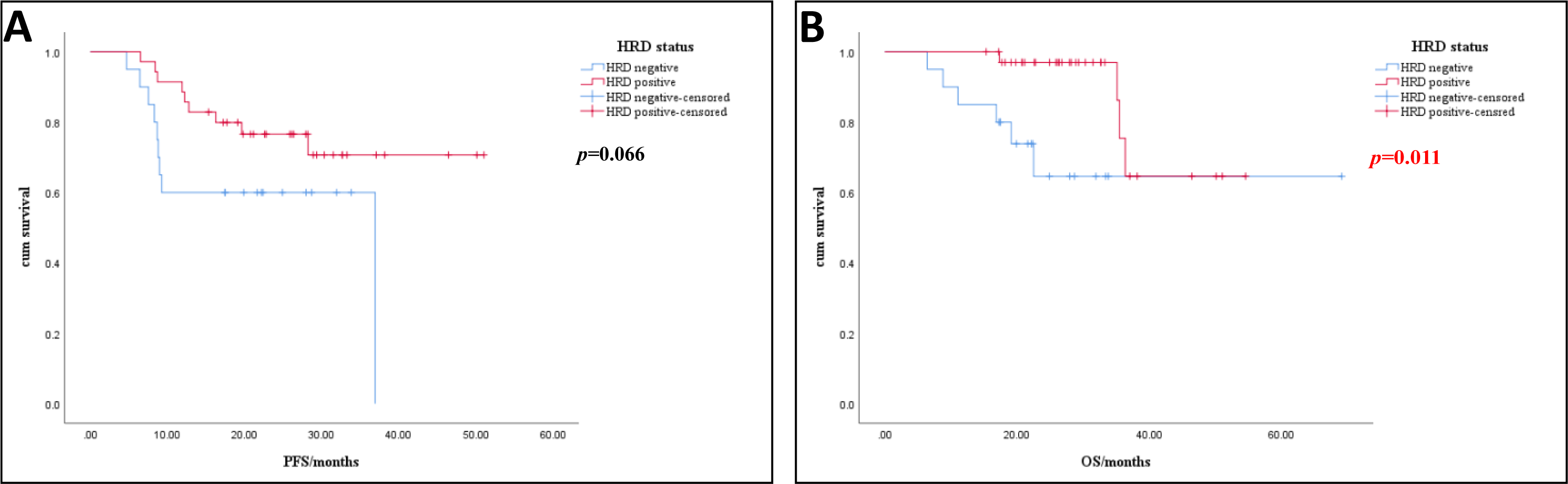

Supplement: Supplementary file 3 — Additional file 3: Supplementary Fig. 3. K-M analysis for study population according to HRD status. (A) PFS by HRD status categorized as positive vs. negative. (B) OS by HRD status categorized as positive vs. negative. [file 12957_2024_3308_MOESM3_ESM.tif]

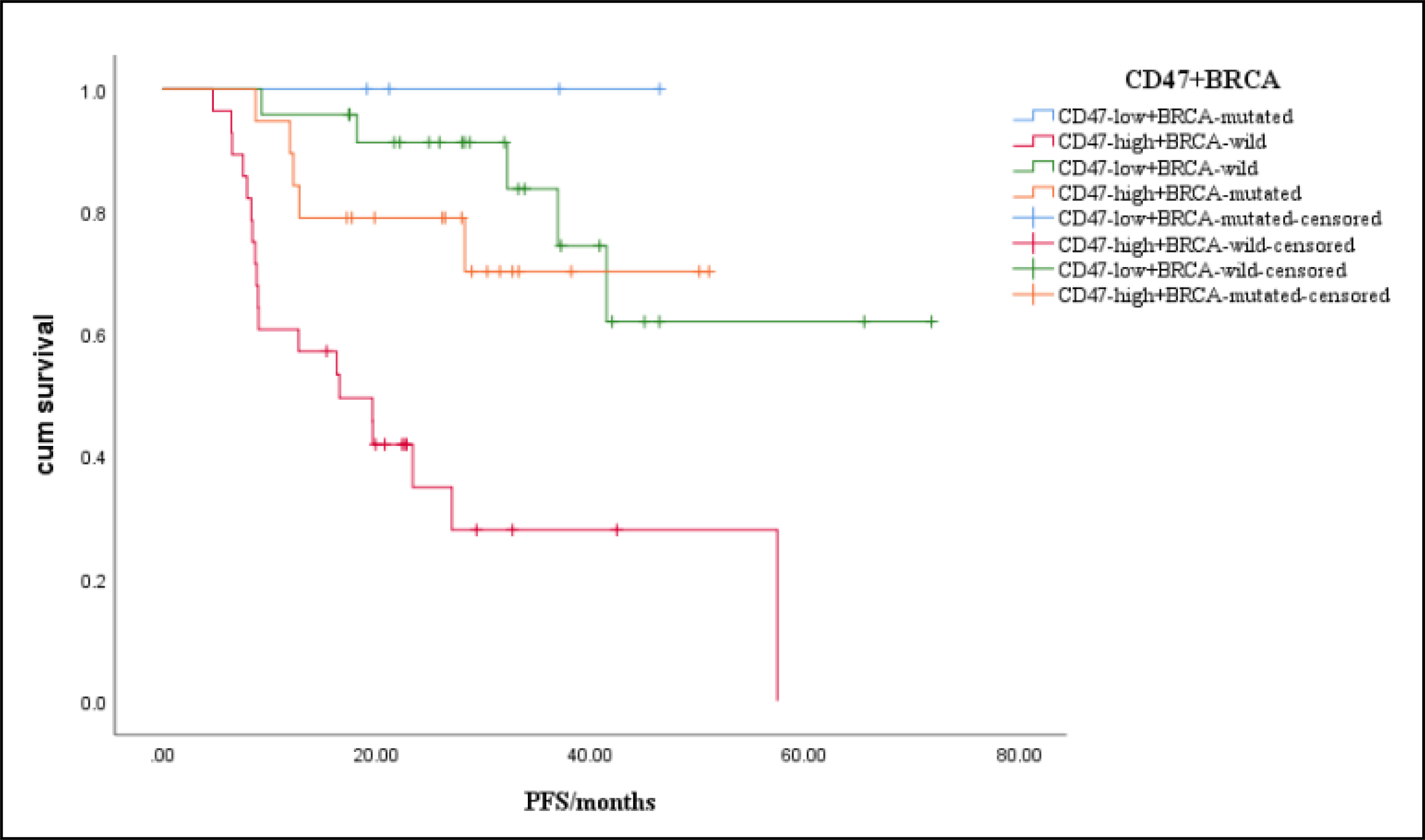

Supplement: Supplementary file 4 — Additional file 4: Supplementary Fig. 4. K-M analysis for the subgroup according to the combination of CD47 expression and BRCA mutation. PFS by the combination of CD47 expression and BRCA mutation status categorized as CD47-high+BRCA-wild subgroup vs. CD47-low+BRCA-mutated, p=0.470; CD47-low+BRCA-wild, p<0.001; and CD47-high+BRCA-mutated subgroups, p=0.006, respectively. [file 12957_2024_3308_MOESM4_ESM.tif]

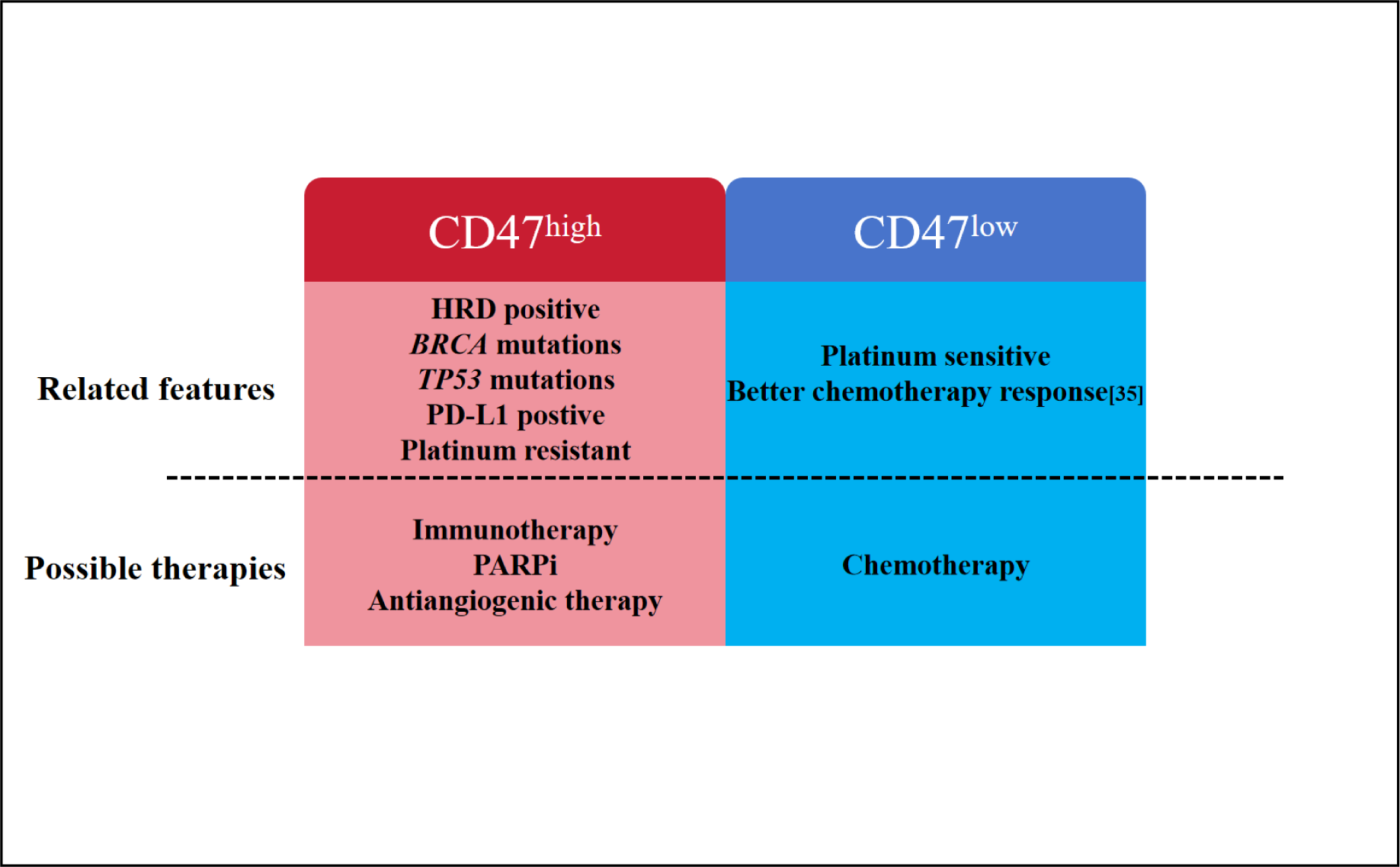

Supplement: Supplementary file 5 — Additional file 5: Supplementary Fig. 5. Possible efficacy therapies in different CD47 expression group. [file 12957_2024_3308_MOESM5_ESM.tif]
