# Supplementary material for: CD47—a novel prognostic predicator in epithelial ovarian cancer and correlations with clinicopathological and gene mutation features
Source: World J Surg Oncol. 2024 Feb 6;22:44. doi: 10.1186/s12957-024-03308-6 (PMC10845810; doi:10.1186/s12957-024-03308-6)
Supplement: Supplementary file 6 — Additional file 6: Supplementary Table 1. Patients’ IHC semi-quantitative score of CD47 expression, CA125 range intervals, and BRCA mutation status. Supplementary Table 2. Relationship between CD47 and platinum status in recurrent subgroup. [file 12957_2024_3308_MOESM6_ESM.docx]

**Supplementary Table 1.** Patients’ IHC semi-quantitative score of CD47 expression, CA125 range intervals, and *BRCA* mutation status

| Case number | CD47 expression score | CA125 value range^*^ | *BRCA* mutation status^†^ |
| --- | --- | --- | --- |
| 1 | 0 | 0 | 0 |
| 2 | 9 | 2 | 1 |
| 3 | 0 | 1 | 0 |
| 4 | 6 | 2 | 1 |
| 5 | 2 | 2 | 1 |
| 6 | 1 | 1 | 0 |
| 7 | 1 | 2 | 0 |
| 8 | 6 | 1 | 0 |
| 9 | 1 | 1 | 0 |
| 10 | 4 | 0 | 0 |
| 11 | 8 | 1 | 1 |
| 12 | 8 | 2 | 0 |
| 13 | 0 | 1 | 0 |
| 14 | 4 | 2 | 1 |
| 15 | 6 | 2 | 0 |
| 16 | 12 | 1 | 0 |
| 17 | 1 | 0 | 0 |
| 18 | 6 | 1 | 0 |
| 19 | 8 | 2 | 1 |
| 20 | 12 | 2 | 1 |
| 21 | 9 | 2 | 1 |
| 22 | 12 | 1 | 0 |
| 23 | 9 | 2 | 1 |
| 24 | 6 | 2 | 1 |
| 25 | 12 | 1 | 1 |
| 26 | 12 | 2 | 1 |
| 27 | 12 | 2 | 1 |
| 28 | 4 | 2 | 0 |
| 29 | 6 | 2 | 0 |
| 30 | 1 | 1 | 0 |
| 31 | 4 | 0 | 0 |
| 32 | 1 | 2 | 0 |
| 33 | 2 | 1 | 0 |
| 34 | 1 | 1 | 0 |
| 35 | 12 | 2 | 0 |
| 36 | 12 | 2 | 0 |
| 37 | 6 | 1 | 0 |
| 38 | 2 | 0 | 0 |
| 39 | 12 | 2 | 0 |
| 40 | 12 | 2 | 0 |
| 41 | 12 | 2 | 0 |
| 42 | 12 | 2 | 0 |
| 43 | 12 | 2 | 0 |
| 44 | 12 | 2 | 1 |
| 45 | 9 | 2 | 1 |
| 46 | 8 | 1 | 0 |
| 47 | 2 | 2 | 0 |
| 48 | 12 | 1 | 0 |
| 49 | 9 | 1 | 0 |
| 50 | 1 | 1 | 0 |
| 51 | 9 | 2 | 0 |
| 52 | 12 | 1 | 1 |
| 53 | 6 | 1 | 0 |
| 54 | 12 | 2 | 0 |
| 55 | 2 | 1 | 0 |
| 56 | 8 | 1 | 1 |
| 57 | 1 | 1 | 0 |
| 58 | 2 | 1 | 0 |
| 59 | 2 | 1 | 0 |
| 60 | 6 | 1 | 1 |
| 61 | 6 | 2 | 0 |
| 62 | 6 | 2 | 0 |
| 63 | 8 | 1 | 0 |
| 64 | 8 | 2 | 0 |
| 65 | 6 | 2 | 0 |
| 66 | 4 | 1 | 1 |
| 67 | 9 | 1 | 1 |
| 68 | 9 | 1 | 1 |
| 69 | 1 | 1 | 0 |
| 70 | 2 | 1 | 0 |
| 71 | 6 | 1 | 1 |
| 72 | 8 | 1 | 0 |
| 73 | 12 | 1 | 0 |
| 74 | 4 | 0 | 1 |
| 75 | 1 | 0 | 0 |

**^*^**: 0, CA125<35U/ml; 1, 35≤CA125<500U/ml; 2, CA125≥500U/ml; ^†^: 0, wild type; 1, pathogenic mutation.

**Supplementary Table 2.** Relationship between CD47 and platinum status in recurrent subgroup.

| Characteristics | Cases | CD47 expression | | *p*-Value |
| --- | --- | --- | --- | --- |
|  |  | Low [cases (%)] | High [cases (%)] | *-* |
| Platinum status | 29 | - | - | 0.027 |
| Sensitive | 16 | 5 (31.3) | 11 (68.7) | - |
| Resistant | 13 | 0 (0.0) | 13 (100.0) | - |
